# C-854-03. Enhancing Patient Safety Through Effective Transfer of Accountability in Burn Care: A Multistage Handover Approach

**DOI:** 10.1093/jbcr/irag033.079

**Published:** 2026-04-06

**Authors:** Anita Au, Shabana Lalji, Gavin Shantz, Alan D Rogers, Stephanie A Mason, Rimona Natanson, Jasmine Segal, Leslie Montgomery, Kristine Laing, Ashley Callahan

**Affiliations:** Sunnybrook Health Sciences Centre, Toronto, ON; Sunnybrook Health Sciences Centre, Toronto, ON; Sunnybrook Health Sciences Centre, Toronto, ON; Ross Tilley Burn Centre, Toronto, ON; University of Toronto, Toronto, ON; Sunnybrook Health Sciences Centre, Toronto, ON; Sunnybrook Health Sciences Centre, Toronto, ON; Sunnybrook Health Sciences Centre, Toronto, ON; Ross Tilley Burn Centre, Toronto, ON; Ross Tilley Burn Centre, Toronto, ON

## Abstract

**Introduction:**

Burn care involves complex, high-risk interventions delivered by multidisciplinary teams across multiple care settings. Effective transfer of accountability (TOA) at key transition points is critical to ensure patient safety, continuity of care, and optimal outcomes. However, inconsistent or unstructured handovers can lead to communication failures and adverse events. This quality improvement project aimed to evaluate and optimize TOA practices across the burn care continuum by implementing a structured, burn-specific handover model at five critical transitions.

**Methods:**

A descriptive qualitative design was used to assess TOA processes at a specialized burn centre. Data were collected through direct observation of handovers, semi-structured interviews with nurses and allied health professionals, and review of relevant documentation tools. Thematic analysis was conducted to identify strengths, gaps, and improvement opportunities. Five key TOA stages were targeted:

1. Pre-admission Handover (EMS to Burn Team): Utilized a standardized form aligned with Advanced Burn Life Support (ABLS) and integrated ambulance documentation to improve early communication of injury severity and pre-hospital interventions.

2. Perioperative Handover (Surgical to Bedside Team): Structured verbal handoffs ensured clear transfer of intraoperative findings, airway status, fluid management, and postoperative plans.

3. Intra-unit RN Shift Handover: Using SBAR framework with emphasized burn-specific assessment, wound care updates, pain management, and psychosocial concerns.

4. Interdisciplinary Team Rounds: Daily multidisciplinary burn team briefings supported shared goals, clinical updates, and proactive care planning across disciplines.

5. Transition to Rehabilitation: Discharge handovers employed an SBAR framework, ensuring continuity in functional status, wound care needs, and psychosocial supports.

**Results:**

TOA practices varied in consistency across transition points. Standardized tools (ABLS-based forms, SBAR templates) improved completeness and clarity. Structured perioperative handovers enhanced care continuity, while RN shift reports showed variability in burn-specific detail. Interdisciplinary huddles fostered collaborative care but lacked standardization. Rehabilitation transitions using SBAR improved communication but required further customization for burn-specific information.

**Conclusions:**

Implementing a structured, multistage TOA model tailored to burn care improves communication, reduces information loss, and enhances patient safety during transitions.

**Applicability of Research to Practice:**

This model underscores the need for discipline-specific TOA tools, staff education on handover best practices, and regular audit mechanisms. Embedding standardized, burn-focused TOA processes into daily practice can lead to safer, more coordinated care across the burn recovery trajectory.

**Funding for the Study:**

N/A.

